# Classification and Definitions of Compensatory Protective Step Strategies in Older Adults: A Scoping Review

**DOI:** 10.3390/jcm13020635

**Published:** 2024-01-22

**Authors:** Maria Melo-Alonso, Alvaro Murillo-Garcia, Juan Luis Leon-Llamas, Santos Villafaina, Mari Carmen Gomez-Alvaro, Felipe Alejandro Morcillo-Parras, Narcis Gusi

**Affiliations:** 1 Physical Activity and Quality of Life Research Group (AFYCAV), Facultad de Ciencias del Deporte, Universidad de Extremadura, 10003 Caceres, Spain; mmeloa@unex.es (M.M.-A.); alvaromurillo@unex.es (A.M.-G.); leonllamas@unex.es (J.L.L.-L.); svillafaina@unex.es (S.V.); maricarmengomezal@unex.es (M.C.G.-A.); felipealmp@unex.es (F.A.M.-P.); 2 International Institute for Innovation in Aging, Universidad de Extremadura, 10003 Caceres, Spain

**Keywords:** postural balance, falls, gait, stepping, elderly, movement, perturbation

## Abstract

Background: The risk for an unexpected fall can be due to increasing age, health conditions, and loss of cognitive, sensory, or musculoskeletal functions. Falls have personal and economic consequences in many countries. Different disturbances can occur during gait, such as tripping, slipping, or other unexpected circumstances that can generate a loss of balance. The strategies used to recover balance depend on many factors, but selecting a correct response strategy influences the success of balance recovery. Objectives: (1) To collect and clarify the definitions of compensatory protective step strategies to recover balance in older adults; (2) to identify the most used methods to induce loss of balance; and (3) to identify the most used spatiotemporal variables in analyzing these actions. Methods: The present review has followed the PRISMA guideline extension for Scoping Review (PRISMA-ScR) and the phases proposed by Askery and O’Malley. The search was conducted in three databases: PubMed, Web of Science, and Scopus. Results: A total of 525 articles were identified, and 53 studies were included. Forty-five articles were quasi-experimental studies, six articles were randomized controlled trials, and two studies had an observational design. In total, 12 compensatory protective step strategies have been identified. Conclusions: There are 12 compensatory protective step strategies: lowering and elevating strategy, short- and long-step strategy, backward and forward stepping for slip, single step, multiple steps, lateral sidesteps or loaded leg sidestep unloaded leg sidestep, crossover step (behind and front), and medial sidestep. To standardize the terminology applied in future studies, we recommend collecting these strategies under the term of compensatory protective step strategies. The most used methods to induce loss of balance are the tether-release, trip, waist-pull, and slip methods. The variables analyzed by articles are the number of steps, the acceleration phase and deceleration phase, COM displacement, the step initiation or step duration, stance phase time, swing phase time and double-stance duration, stride length, step length, speed step, speed gait and the type of step.

## 1. Introduction

It is known that the concept of fall has different definitions; however, the definition most used is provided by the Prevention of Falls Network Europe group (ProFaNE), “an unexpected event in which the participants come to rest on the ground, floor, or lower level” [1]. A fall can be experienced by all people, but the probability of falling increases with age [2]. The risk for unexpected falls can be due to increasing age, loss of cognitive and musculoskeletal function, and presenting health problems [3]. Falls have personal and economic consequences in many countries [2,4]. In this sense, adults over the age of 65 will suffer at least one fall per year and after the age of 75, the probability of falling increases [4]. Falls can have health consequences, such as fractures or head injuries [2], and attending to these injuries has a high economic impact [5,6]. In addition, these injuries cause hospitalizations and premature admissions to nursing homes, which can have negative and detrimental effects on the quality of life for older adults [2].

Falls can occur outdoors or indoors, but they present the same probability for causing injuries [7]. In addition, falling or even fear of falling can generate changes in gait pattern, decreased mobility, reduced social contact, and impaired ability to perform daily living activities [8,9]. On the other hand, falls reported by older adults usually occur while walking, stepping over a curb or step, and going up or down stairs [7,10]. Thus, two types of factors can produce a fall: environmental factors such as outdoor objects or walking surface conditions and factors related to the activity or the behavior of the person at the time of the fall [11].

Gait is a skill coordinated by the nervous system using the perceived multisensory information and allows moving the body from one point to another through cyclical movements of the upper and lower body. In addition, the gait pattern is constantly being adjusted to maintain balance and adapt to the factors of the surrounding environment [12]. Different disturbances can occur while walking, for example stumbling or slipping, which can cause a loss of balance. In this sense, the central nervous system uses two types of postural adjustments to maintain the center of mass (COM) within the base of support (BOS): anticipatory postural adjustments and compensatory postural adjustments [13]. Postural adjustments are developed to recover balance through different strategies. The use of each strategy depends on the postural stability, the reaction time, and the phase of the cycle [14].

Numerous studies have analyzed gait in older adults and its relationship to fall risk. Different spatiotemporal variables related to the risk of falling have been identified in the scientific literature, such as speed gait [15,16,17,18], stride length [19], stride speed, double support time, stride width, stride time [18], or swing time variability [18,19]. The analysis of these parameters offers information on gait variability and its relationship with risk of falling. Thus, an increased age is associated with loss strength, decreased mobility, and postural control, that can influence gait performance and risk of fall. The gait in older adults is characterized by a decrease in step length, the stride length, and walking speed, and by the increase in step time, stride time, swing time, and the double support time [20]. Thus, older adults present a cautious and slower gait, which is a predictor of motor disability in activities of daily living, mortality, and cognitive decline (memory, executive function, and global cognition) [15,16,17].

The analysis of spatiotemporal variables of gait has been carried out under normal conditions [20]. In addition, it is important to know how to resolve motor problems in the presence of external perturbations. To answer this question, scientists have simulated perturbations in laboratory conditions to analyze the compensatory protective step strategies [21,22,23,24], terms suggested by Tisserand et al. [13]. In this sense, different instruments have been used to train balance [25] or generate falls such as the waist-pull [22], mobile platforms [26,27], slippery floor surfaces [28], release system [29,30] or new robotic devices [31]. Currently, there are also new devices that assess postural control during static and dynamic ways. These types of devices help to assess the risk of falling in a more objective form due to the precision in the analysis of the postural control movement [32]. Additionally, new assistive robots have been developed to improve stability control in walking, and could be very useful in reducing the risk of falling [33]. On the other hand, these studies analyze the strategies used to recover balance by older adults in the presence of the external disturbances mentioned above. The selection of a correct response strategy can prevent a fall and its consequences.

Since there are several definitions of compensatory protective step strategies in the literature, as well as different perturbation methods and spatiotemporal variables used for the analysis, this scoping review aims to collect and clarify the definitions of compensatory protective step strategies to recover balance in older adults. Specifically, all the strategies that involve a change in BOS [13] will be compiled in this scoping review studying the different perturbation methods and spatiotemporal variables described by the scientific literature.

## 2. Materials and Methods

The current review followed the PRISMA guideline extension for Scoping Reviews (PRISMA-ScR) [34] and the phases proposed by Askery and O´Malley [35]: (1) identify the research questions; (2) identify relevant studies; (3) study selection; (4) charting the data; and (5) collate, summarize and report the results. To answer these questions, the term compensatory protective step strategies suggested by Tisserand et al. [13] has been used as a reference to identify the aim of the present review.

### 2.1. Identify Research Questions

Research questions were developed to identify the research objectives and served to guide the search strategy. The research questions are the following: (a) What compensatory protective step strategies do older people use in response to a loss of balance? (b) What methods of perturbations do researchers use to cause a loss of balance? (c) What are the spatiotemporal variables that studies analyze?

The current review includes studies that analyzed loss of balance during gait in older people. In addition, all disturbance methods were included to identify and collect them.

### 2.2. Identify Relevant Studies

The search was conducted in the following databases: PubMed, Web of Science, and Scopus. The search terms and the Boolean operators used were (ageing OR aging OR older people) AND (trip OR stepping OR step OR walking) AND (fall recovery). The search ended on 7 December 2023 and there was no date restriction on the search of articles. See Table 1 for further details of the PubMed search strategy. The Excel program (Microsoft Office Professional Plus 2019) was used to collect the search results and identify duplicate articles. In the event that an article did not present the full text, the authors were contacted.

### 2.3. Study Selection

Studies were included if they met the following inclusion criteria: (a) articles conducted with older adults, age ≥ 65 years old, (b) articles that analyzed stepping strategies used after a disturbance in a static or dynamic situation, (c) articles with a study design of randomized controlled trials, cohort studies, cross-sectional studies, or clinical trials, and g) articles that studied the gait.

The following exclusion criteria were established: (a) studies not written in English or Spanish, (b) studies of populations with diseases that can affect the gait (Alzheimer’s, Parkinson’s, osteoporosis, stroke, arthritis, multiple sclerosis, cancer in active treatment, or atrophy paralysis), (c) patients with fractures in the lower limbs (hip, knee, and ankle), and (d) studies that were reviews of the literature, articles presented as abstracts in a congress or seminar, clinical guidelines, and study protocols.

### 2.4. Charting the Data

The following data were extracted: title, authors, publication year, research objectives, population, study design, methodology, name of strategies, and definitions. The full texts were reviewed by two authors.

The search was conducted by M.M.-A. and checked by A.M.-G. In case of disagreement, a consensus discussion directed by J.L.L.-L. was performed. The search ended on 7 December 2023.

### 2.5. Collate, Summarize and Report the Results

Firstly, a descriptive analysis of articles included in the present review was carried out. This analysis included the type of the study, the aim of the study, the perturbation method, and instrument used for it, the type of compensatory protective step analyzed, and the variables and the measuring instruments. Secondly, the definitions used by the authors for each strategy identified were reported and grouped according to the type of disturbance.

## 3. Results

### 3.1. Article Selection

**Figure 1:**
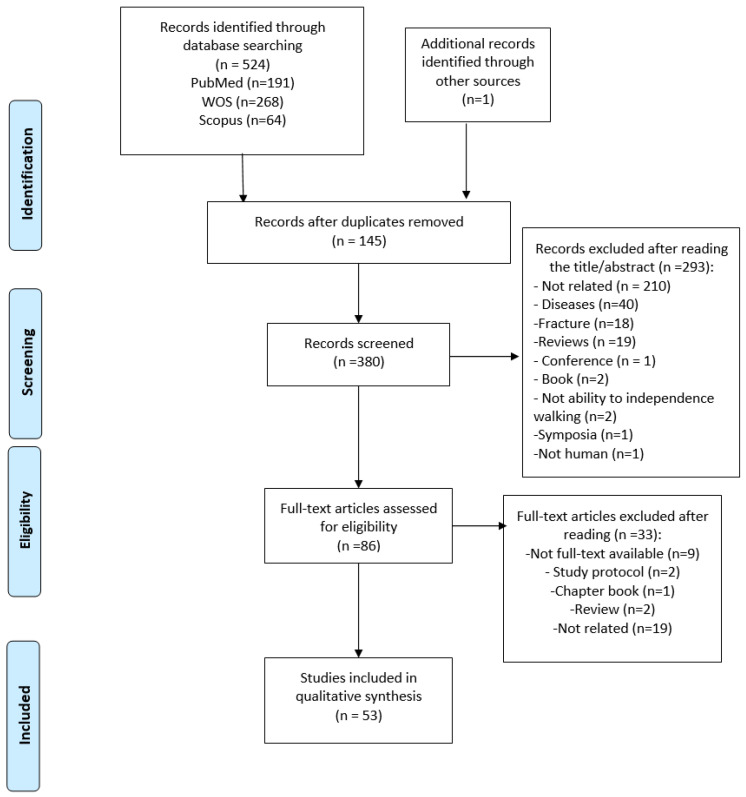
Flow chart for selection of studies.

Figure 1 shows the article selection process followed in the present scoping review. A total of 524 articles were identified in electronic databases: PubMed (191 articles), WOS (268 articles), and Scopus (64 articles). In addition, one article was identified through other sources. After that, 145 articles were removed because they were duplicated studies. Moreover, 293 articles were removed after reading title/abstract (see Figure 1 for reasons). Of the remaining 86 articles, 33 were removed (see Figure 1 for reasons). Finally, after this exhaustive selection, 53 articles were included.

### 3.2. Study Design and Methods of Perturbations

Forty-five articles included were quasi-experimental studies [21,22,23,24,26,27,28,29,30,31,36,37,38,39,40,41,42,43,44,45,46,47,48,49,50,51,52,53,54,55,56,57,58,59,60,61,62,63,64,65,66,67,68,69,70], six articles were randomized controlled trials studies [71,72,73,74,75,76], and two article were observational studies [77,78].

The included studies used different perturbation methods and all these methods are reported in Supplementary Materials (Table S1). The method most used was the tether-release method, reported in 14 studies [29,30,39,40,41,43,45,46,51,52,60,61,62,70]. Also, the trip method was reported in 13 studies [21,31,39,42,54,55,56,62,64,69,71,72,74] and the surface translation method was reported in 12 studies [24,26,36,37,44,49,53,62,72,73,75,77]. Other methods reported were the waist-pull method used by 11 articles [22,23,38,48,49,50,57,66,67,73,76], the slip method used by 8 articles [27,28,63,64,65,71,74,79], the perturbing shoes method used by 2 articles [31,47], the perturbations of the support surface used in one article [59], and only one article used a video camera [78].

### 3.3. Types of the Compensatory Protective Stepping Strategies

**Figure 2:**
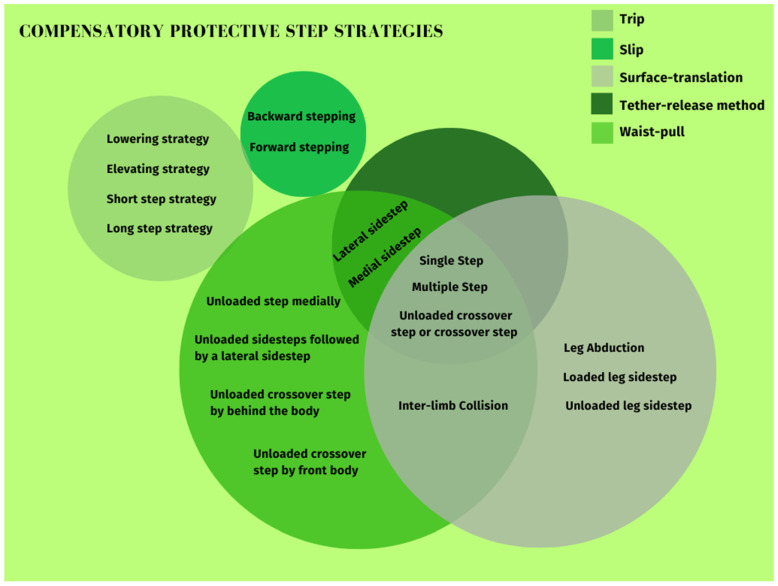
Types of compensatory protective step strategies depending on the perturbation method.

All the types of compensatory protective step strategies found on the included studies were compiled and reported in Table 2. In addition, a schematic Venn diagram of the compensatory protective step strategies collected by each perturbation method was included in Figure 2. There are two methods that have no strategy in common (trip and slip methods) with the others and the rest have some strategies in common; these strategies are represented within two or more circles that share those same strategies.

The most repeated strategies for disturbance by method of travel were the descent and elevation strategy [31,55,64,69,74]. For the slip disturbance method, these two strategies were also reported [74]. For the waist-pull disturbance method, the strategies reported were the lateral sidestep, the unleaded leg sidestep, the loaded leg sidestep, the crossover step, and the collision between feet [23,38,50,66,67]. For the tether-release methods, the strategies of single and multiple steps were reported, principally [29,30,39,41,45]. For the surface translation methods, the strategies reported were the single and multiple steps, the loaded leg sidestep, the unloaded leg sidestep, the crossover step, and hip abduction [24,37,77].

## 4. Discussion

### 4.1. Principal Findings

The aims of the present scoping review were to compile all the compensatory protective step strategies for loss of balance in older adults and to identify perturbation methods and spatiotemporal variables used to analyze the collected strategies. This review included 53 articles, 45 of them were quasi-experimental studies [21,22,23,24,26,27,28,29,30,31,36,37,38,39,40,41,42,43,44,45,46,47,48,49,50,51,52,53,54,55,56,57,58,59,60,61,62,63,64,65,66,67,69,70], 6 articles were randomized controlled trial studies [71,72,73,74,75,76], and 2 articles were observational studies [77,78].

In recent years, the number of studies on step strategies have increased due to the importance of accurately stepping and avoiding or passing an obstacle in daily life activities [80]. Although some studies analyze different step strategies [23,31,37,53], no review has been found that compiles all the step strategies described in the literature. However, this scoping review offers a list of all the strategies identified in recent years and their different definitions.

The included studies have used different perturbation methods to produce strategies. The main methods reported were the tether-release method (in 14 studies), the trip method (in 13 studies), the surface translation method (in 12 studies), the waist-pull method (in 11 studies), and the slip method (in 8 studies). The most repeated strategies for the trip method were the lowering and elevation strategies. The articles that analyze both strategies (lowering and elevation strategy) [31,55,64,69,74] did not find differences between using one or another, but it could be a determining factor for the success of the recovery. However, there are differences in the analyses between healthy older adults and young adults. Older adults had less of a proper placement of the recovery limb to recover balance which could make older adults fall more [55]. Furthermore, older adults faller have a greater center of mass (COM) velocity and larger trunk flexion compared to older adults [69], increasing the risk of falling.

On the other hand, there are two other strategies with the trip perturbation method, such as short- and long-step strategies, but only one article mentioned it [21]. Older and young adults used the short-step strategy when they had a short time to avoid the obstacle. This strategy changes when there is more time, using the long-step strategy. The short-step strategy is commonly used by older adults because they feel that it is easier, representing a conservative action. Although the long-step strategy is safer when there is more time to choose the strategy, it is more difficult to execute since the walking speed and the distance of the obstacle must be considered [21]. Although there is not much information on these strategies, the information provided in this study [21] reveals the importance of working on the capacity to make decisions with or without a time deficit to improve step precision as a function of walking speed.

For the slip methods, there are two strategies: backward and forward slips [74]. These strategies can be performed using one-foot (split slip) or two-foot (feet-forward slip) methods, depending on the recovery foot placement [27]. Slip analysis is unaffected by the slow gait velocity short-step length, age, deterioration of muscular strength, and sensory degeneration [28], but recovery foot placement can affect the capacity to regain balance [58]. The slip with the two-foot method has less fall risk compared to the one-foot method. However, the slip with the two-foot method is associated with more serious injuries because the COM velocity is higher as well as is the influence of having a greater angle of the trunk being backward [27]. It is difficult to indicate which is best or most appropriate for older adults.

For the tether-release methods, the main strategies analyzed are single and multiple steps [29,39,41]. The analysis of these strategies indicated that older adults often use the multiple step [41,60] unlike young adults [60]. This may be due to reasons such as older adults need more time to stop the COM progression (few control postural) [51,60], they have less movement amplitude [46,51,60], they have shorter step length compared to young adults, the velocity movement of the lower limb is slower [30,60,61], the lower limbs’ eccentric muscle work capacity is not effective enough to absorb the momentum of the fall with a single step [51], and the muscle activation lowers with multiple steps [43]. Age and all these reasons might cause older adults to not regain balance with a single step. A single step strategy should be the most used, instead of multiple steps, since being able to take a quicker and larger step helps to maintain the control of COM [51]. Some studies have demonstrated that disturbance training can change the type of strategies, stop using multiple steps, and start to more often using single steps [41].

On the other hand, with the waist-pull method and surface translation method, it is possible to study more strategies such as lateral steps, single step, multiple steps, and collision between feet and leg abduction [23,24,50]. Thus, to perform the step analysis, force platforms, motion capture systems, instrumented walkway systems, or reflective markers were used to obtain different variables. Some studies indicated that older adults often used more unloaded crossover steps (UCS) [37,50], multiple steps, and had more collision between feet [50] than young adults; the young adults use loaded sidestep (LSS). The UCS and multiple step strategies are more complex and dangerous to recovery balance than LSS, because there are a greater number of collisions between feet [38,50,67]. The UCS or unloaded sidestep (USS), cross step front, cross step back and medial step are less-efficient strategies and safer than using LSS for older adults. However, using UCS has some advantages such as shorter step initiation time. However, there are disadvantages to consider, such as increased time in the stance phase of a leg, greater instability, and it requires a longer step length, as well as it is associated with an increased collision between feet [50]. The older adults choose this strategy because conducting LSS is more complex and difficult for them, although it is safer. LLS is more difficult due to neuromuscular factors and aging [38,50], and because LSS is characterized by having a larger COM velocity [23,38,50].

This scoping review recommends the analysis of reaction time, step time, stance phase time, swing phase time, and double-stance duration, stride length [19], step length, speed step, speed gait [15,16,17,18], and the type of step. At least the analysis of the above variables is recommended due to their relationship with the risk of falling, already demonstrated in the analysis of normal gait without external disturbance [20]. In addition, with the present scoping review, it is recommended to continue studying the relationship between compensatory step strategies and the risk of falling, including the above variables. For analysis of single step, multiple steps, lateral steps, and collision between feet strategies, the waist-pull and surface translation methods were mainly used. Generally, the spatiotemporal variables studied in these compiled strategies are reaction time, stride length, step length, step width, step time, number of recovery step, type of step, gait speed, COM position, or COM position relative and trunk angle.

In total, 61 definitions of step strategies have been collected, of which 10 are repeated. The rest of them describe the step strategies differently, although several of them are similar. The current scoping review recommends reading each of the definitions of the strategies given by each author to select the appropriate ones for future research. Furthermore, 14 strategies were identified by this review. Only 12 of them are included in the group of compensatory protective step strategies because they meet the criteria of Tisserand et al. [13] and they are characterized by the fact that participants take a step to recover balance. The included strategies are lowering and elevating strategy, short- and long-step strategy, backward and forward stepping for slip, single step, multiple steps, lateral sidesteps, or loaded leg sidestep unloaded leg sidestep, and crossover step (behind and front) and medial sidestep. The leg collision is not included because it was considered an external disturbance. In addition, in the definitions offered by authors for leg collision and hip abduction, there is not an explanation of the balance recovery with the execution of a new step.

In view of the previous paragraphs, the compensatory protective step strategies are an important topic to be further investigated by the different professionals to reduce the risk of falling [67]. It is necessary that older adults choose the strategies that are safer and more effective to balance recovery. In this regard, older adults should stop using multiple steps to use single step or LSS. Therefore, we can create specific training exercises to induce these changes. Previous studies have shown that reactive balance or disturbance training improves reaction times [73], reduces COM velocity [69], and trunk flexion [71,72]. Furthermore, it would be recommendable to train lower body strength. This training should be focused on improving the strength of the knee extensor and ankle dorsiflexor muscles [41], or strength training at high speeds to improve muscle activation. Also, it would be relevant to improve flexibility, range of movement, COM control [51,60], and the margin of stability [41], as well as teaching the slowing down of COM displacement and trunk flexion. As a result of these improvements, older adults will be able to place their limb in a more correct position and be able to apply sufficient force to avoid a fall. In addition, introducing specific trainings should be introduced to improve the motor control and adaptability of the person, helping to make a faster and more optimal motor decision depending phase [74]. These are some proposals that can be carried out by physiotherapists, researchers, occupational therapists, or physical educators to reduce the risk of falling in older adults. In this regard, a previous study has shown that specific training reduces the risk of falling by up to 51% [74].

### 4.2. Comparison to Literature

Maintaining balance is a complex task and researchers have a high interest in knowing and understanding how the organism solves an unbalance to avoid a fall. The review by Jacobs et al. [81] explained that a fast response is required to solve the motor problem. A fast and automatic postural response is sometimes influenced by the involvement of the cerebral cortex to select the optimal and appropriate response. The involvement of the cerebral cortex influences in response time, which is longer, and its activation depends on the cognitive state, sensorimotor conditions, previous experiences, and characteristics of the perturbations [81]. Tisserand et al. [13] agrees with Jacobs and Horak [81] and in addition indicated the adjustments involved in the task. In this sense, the central nervous system (CNS) is responsible for maintaining static and dynamic balance, using two types of postural adjustments: anticipatory postural adjustment (APA) and compensatory postural adjustment (CPA). These postural adjustments generate movements to recover balance: fixed support strategies or change support strategies. Moreover, both reviews explain what happens in the event of a loss of balance and what systems are activated to resolve a loss of balance. However, previous reviews [13,81] did not identify the strategies that are used to recover balance during gait. On the other hand, the review by Potocanac et al. [80] mentions some of the strategies collected in this scoping review, but does not classify them into groups.

### 4.3. Strengths and Limitations

At first, the search for the current scoping review was developed in different databases until December 2023. The present review included a large number and variety of studies with different perturbation methods and instruments which cause a fall. Another strength of this study is the compilation of a step strategy list with their different definitions. Moreover, the spatiotemporal variables used to analyze compensatory protective step strategies were also identified and compiled.

One of the limitations of the present study is the exclusion of articles found in the gray literature (e.g., conference abstracts, book chapters or research reports), the exclusion of studies not published in English or Spanish, the not having access to free text articles, and the lack of searches in relevant databases such as EMBASE, Cochrane, CINAHL, and PEDro. Therefore, some articles that can be included in this review might not be included. In this regard, there are nine articles for which we have not found the full text. Authors were contacted via ResearchGate and email, but as of the date of publication of this article, no author has responded. In addition, it is important to mention that some authors did not include the definitions of the step strategies, which can make it difficult to classify them. In this sense, based on this study, we recommend that the definitions of the step strategies should be included in future research. Lastly, the risk of bias and the methodological quality of the included articles have not been analyzed. The PRISMA guidelines for scoping reviews stated that these issues are not applicable for this type of review. In addition, the heterogeneity in terms of design and the quantity of articles included in this review would probably make this analysis incomprehensible.

### 4.4. Future Research

This scoping review recommends that future research clarify what types of strategies are going to be studied and to define them correctly throughout the study. Furthermore, this review recommends that the description be carried out using the definitions collected in the current review to help identify strategies and reduce heterogeneity.

Another recommendation for future studies is that researchers include the analysis of step strategies under the dual task paradigm, since the activities of daily living are presented as a combination of two or more tasks at the same time [82,83] and this can affect the performance of one or both tasks [84,85]. Research under the dual task paradigm will facilitate the study of the effectiveness of strategies under different conditions. On the other hand, it is also interesting to increase knowledge about the step strategies of people with motor pattern problems and their relationship with the risk of falling.

## 5. Conclusions

Based on the current literature, this scoping review provided a summary compiling and clarifying definitions of compensatory protective step strategies for recovering balance in older adults. In addition, the most commonly used methods to induce the loss of balance and the most used spatiotemporal variables in analyzing these actions were collected.

In this sense, there are 12 compensatory protective step strategies: lowering and elevating strategy, short- and long-step strategy, backward and forward stepping for slip, single step, multiple steps, lateral sidesteps or loaded leg sidestep unloaded leg sidestep, crossover step (behind and front), and medial sidestep. To standardize the terminology applied in future studies, we recommend collecting these strategies under the term of compensatory protective step strategies.

The most used methods to induce loss of balance are the tether-release, trip, waist-pull, and slip methods. The variables analyzed by articles are the number of steps, the acceleration phase and deceleration phase, COM displacement, the step initiation or step duration, stance phase time, swing phase time and double-stance duration, stride length, step length, speed step, speed gait, and the type of step.

To reduce heterogeneity in future research, it is recommended to define and identify the strategies following the definitions compiled in this review. In addition, it is also recommended to read this review to learn about the perturbation methods used by researchers and the spatiotemporal variables analyzed to study each step strategy. Although this review aims to standardize this field of knowledge, the conclusions must be taken with caution due to the heterogeneity found in this review.

## Figures and Tables

**Table 1:** **PubMed database search strategy.**

**Date:** 7 December 2023	
(Ageing OR aging OR older people) AND (trip OR stepping OR step OR walking) AND (fall recovery)	
Records screened	191
Records excluded (literature reviews, not full-text articles, guideline reports, study protocol, or conference abstract)	20
Full-text articles excluded (exclusion criteria)	28
Full-text articles excluded (inclusion criteria)	106
Articles included	37

**Table 2:** **Name and definition of the different types of compensatory protective step strategies.**

Method Perturbation	Author (Year)	Name of Strategy	Definition
Trip	Wang Y. et al. (2020) [64]	Lowering strategy	The obstructed foot was quickly lowered to the ground and the contralateral unobstructed foot took a recovery step.
		Elevating strategy	The obstructed foot took a recovery step after hitting the obstacle.
		Obstacle crossing	Crossing over the obstacle without hitting it.
	Wang S. et al. (2023) [69]	Lowering strategy	Consists of lowering the tripped limb behind the obstacle and taking a recovery step with the other limb.
		Elevating strategy	Consists of taking a recovery step by lifting the tripped foot over the obstacle.
	Pijnappels et al. (2005) [55]	Elevating strategy	This strategy is observed after a perturbation in early swing and consists of an elevation of the obstructed swing limb to overtake the obstacle.
		Lowering strategy	This strategy is observed during late swing and consists of an immediate placement of the obstructed foot on the ground, followed by a step of the contralateral limb to overtake the obstacle.
	Okubo et al. (2019) [74]	Elevating strategy	The obstructed limb cleared the obstacle after obstacle contact.
		Lowering strategy	The obstructed limb was quickly lowered to the ground before the obstacle.
	Pavol et al. (2001) [31]	Elevating strategy or reaching strategy	The tripped limb is used as the recovery limb as the tripped foot is lifted over the obstacle in a continuation of the original step. The contralateral stance limb acts as the support limb during the recovery step. Elevating and reaching strategies are differentiated based on whether recovery limb flexion occurs at multiple joints or primarily at the hip, respectively.
		Lowering strategy	The tripped foot is immediately lowered to the ground on the near side of the obstacle. The tripped limb then acts as the support limb as the contralateral recovery limb executes the initial recovery step across the obstacle.
	Chen et al. (1994) [21]	Short-step strategy (SSS)	To overcome the obstacle, first a shortening of the normal step and then an additional step is taken to cross the obstacle.
		Long-step strategy (LSS)	To overcome the obstacle, a longer step is taken.
Slip	Okubo et al. (2019) [74]	Backward stepping	The first recovery foot landed posterior to the slipping (contralateral) foot.
		Forward stepping	The first recovery foot landed anterior to the slipping (contralateral) foot.
Waist-pull	Yungher et al. (2012) [67]	Lateral sidestep (LSS)	Perform the first step with the passively loaded leg (near side to pull).
		Unloaded crossover step by front body (CSF)	Perform the first step with the passively unloaded leg (far side to pull), in front of the body.
		Unloaded crossover step behind the body (CSB)	Perform the first step with the passively unloaded leg (far side to pull) behind the body.
		Unloaded step medially (MS)	Perform the first step with the passively unloaded leg that moves medially towards, but not past, the passively loaded leg.
		Inter-limb collisions	-
	Young et al. (2013) [66]	Lateral sidestep (LSS)	Perform the first step with a lateral sidestep with the passively loaded leg.
		Unloaded crossover step or crossover step (COS)	Perform the first step with an unloaded crossover step with the passively unloaded leg in front of or behind the body.
		Medial sidestep (MSS)	Perform the first step with an unloaded step with the passively unloaded leg that moves medially towards the passively loaded leg.
	Mille et al. (2005) [50]	Single step	-
		Multiple steps	-
		Loaded sidestep (LSS)	Performing a sidestep with the passively loaded leg.
		Unloaded medial step (UMS)	The passively unloaded limb was moved medially towards the other leg without crossing over the stance leg.
		Unloaded crossover step (UCS)	Performing a crossover step, either in front of or behind the body, with the passively unloaded leg.
		Collision between feet	-
	Borrelli et al. (2019) [38]	Lateral sidestep (LSS)	The COM is moved passively, relative to the BoS, such that the leg contralateral to the direction of the imposed COM movement is passively unloaded while the ipsilateral leg is passively loaded. The step starts with the passively loaded limb.
		Unloaded sidesteps (USS: CSF; CSB; MSS)	The COM is moved passively, relative to the BoS, such that the leg contralateral to the direction of the imposed COM movement is passively unloaded while the ipsilateral leg is passively loaded. The step starts with the passively unloaded limb. Three different initial stepping strategies have been identified: (1) crossover step to the front (CSF); (2) cross-over step to the back (CSB); and (3) medial sidestep (MSS).
		Multiple steps	Take more than one step to recover balance.
		Inter-limb collisions	-
		Single step	Take a step to recover balance.
	Borrelli et al. (2021) [23]	Laterals sidestep (LSS)	The COM is moved passively, relative to the BoS, such that the leg contralateral to the direction of the imposed COM movement is passively unloaded while the ipsilateral leg is passively loaded. The step starts with the passively loaded limb.
		Unloaded sidesteps (USS: CSF; CSB; MSS)	The COM is moved passively, relative to the BoS, such that the leg contralateral to the direction of the imposed COM movement is passively unloaded while the ipsilateral leg is passively loaded. The step starts with the passively unloaded limb. Three different initial stepping strategies have been identified: (1) crossover step to the front (CSF); (2) crossover step to the back (CSB); and (3) medial sidestep (MSS).
		Multiple steps	Take more than one step to recover balance.
		Inter-limb collisions	-
		Single step	Take a step to recover balance.
		Single lateral sidesteps (LSS1)	Take only a step with the passively loaded limb.
		Unloaded sidesteps followed by a lateral sidestep (USS-LSS2)	Take an unloaded sidestep followed by a lateral sidestep.
Tether-release method	Werth et al. (2021) [29]	Single step	Take a step to recover balance or if a follow- up step of the contralateral limb did not exceed the anterior displacement of the recovery limb.
		Multiple steps	Take more than one step to recover balance or if took a contralateral limb exceed the anterior displacement of the recovery limb.
	Wojcik et al. (1999) [30]	Single step	Regain balance with a step.
		Multiple steps failure	These occur when the subject took a second right leg step of any kind or when she took a left leg step whose length exceeded 30% of body length.
	Bosquée et al. (2021) [39]	Single step	Take an only step to recover stability or if a follow-up step of the contralateral limb did not exceed the anterior displacement of the recovery limb’s foot.
		Multiple steps	Take any additional step of the recovery limb.
	Carty et al. (2012) [41]	Single step	Use a single step to recover balance.
		Multiple steps	Use a multiple step to recover balance.
	Graham et al. (2015) [45]	Single step	Take an only step to recover balance.
		Multiple steps	Take a second step of any kind by the stepping limb or anterior progression of the non-stepping foot past the stepping foot following its initial step.
	Tashiro et al. (2021) [70]	Single step	Recover balance with a single step.
		Multiple steppers	Using two or more steps for balance recovery.
		Lateral steppers	A step with passively loaded leg.
		Crossover steppers	A step with passively unloaded leg and past the loaded leg either in front of or behind the body.
		Medial steppers	A step with passively unloaded leg but not past the loaded leg.
Surface translation	Batcir et al. (2020) [37]	Single step	Regain balance with a step.
		Multiple steps	Multiple steps to recovery balance.
		Loaded leg sidestep (LSS)	Perform the first step after the perturbation in the opposite direction of the platform translation.
		Unloaded leg sidestep (ULSS)	Perform the first step in the same direction of the platform translation.
		Crossover step (COS)	Stepping with the unloaded leg in the opposite direction of the platform translation while swinging the leg over the loaded leg.
		Leg abduction	Abducting the unloaded leg and standing on one leg only.
	Batcir et al. (2022) [24]	Single step	Use a single step to recover balance.
		Multiple step	Use more than one step to recover balance.
		Loaded-leg sideway stepping (LLSS)	Perform the first step sideway with the loaded leg after the perturbation.
		Unloaded-leg sideway stepping (ULSS)	Perform the first step sideway with the unloaded leg after the perturbation.
		Crossover stepping (COS)	Perform the first step with the unloaded leg, while crossing the one leg over the other leg.
		Hip abduction	Abducted hip joint of the unloaded leg laterally.
		Leg collisions (Col)	-
	Batcir et al. (2018) [77]	Unloaded leg sidestep (ULSS)	Perform the first step in the same direction of the platform translation.
		Loaded leg sidestep (LLSS)	Perform the first step after the perturbation in the opposite direction of the platform translation.
		Crossover step (COS)	Stepping with the unloaded leg in the opposite direction of the platform translation while swinging the leg over the loaded leg.
		Leg collision (Col)	Leg collision occurs between the swinging leg and the loaded leg.
		Leg abduction (Abd)	Abducting a leg and standing on one leg only.
		Multiple steps	Multiple steps are a balance response that consists of more than one step, whether moving both legs and taking a few steps with the same foot.

Center of mass (COM); base of support (BoS).

## Data Availability

No new data were created or analyzed in this study. Data sharing is not applicable to this article.

## Supplementary Materials

The following supporting information can be downloaded at: https://www.mdpi.com/article/10.3390/jcm13020635/s1, Table S1: Study characteristics: study design, population, perturbation, strategies, and outcomes.
