# Supplementary material for: Classification and Definitions of Compensatory Protective Step Strategies in Older Adults: A Scoping Review
Source: J Clin Med. 2024 Jan 22;13(2):635. doi: 10.3390/jcm13020635 (PMC10816706; doi:10.3390/jcm13020635)
Supplement: Supplementary file 1 [file jcm-13-00635-s001.zip › jcm-2758663-supplementary.pdf]

Table S1. Study characteristics: study design, population.

Author and Year	Study Design and Population	Study Aim	Perturbation Method and Instrument	Compensatory Protective Step Strategies	Variables and Measuring Instrument
Raffegeau et. al. (2019) [56]	Quasi-experimental. n= 54; W (72±5 years) and M (72±5 years).	Examine gender differences in the performance of adaptive locomotor tasks and evaluate potential contributing factors.	Trip: Walking in a walkway 8 meters: forward walking, backwards walking and obstacle crossing.	-	Step width, step speed and step length. Approach distance, toe-clearance and landing distance *(Motion capture technology, reflective marker and video camera).*
Wang Y. et. al. (2020)[64]	Quasi-experimental. n= 40 MW; (67.9 ±5.5 years).	Investigate the extent to which an obstacle-induced trip paradigm in older adults could reduce trip-induced falls.	Trip a walking in a walkway 7 meters. The obstacle of metal turns on with electromagnets.	Lowering strategy. Elevating strategy. Obstacle crossing.	COM position and velocity, pre-post trip *(Force platform)*. Toe clearance, recovery step length, trunk angle *(Camera motion capture system and reflective markers).*
Wang S. et. al. (2017)[27]	Quasi-experimental. n=195 MW ;(72.3±5.3 years).	Investigate whether the recovery foot placement would determine the slip types, the likelihood of fall, and the severity of fall.	Slip was induced by movables platforms, low-friction, in a walkway 7 meters.	-	Deceleration time recovery foot, step time, liftoff and touchdown foot, and trunk angle *(Camera motion capture system and reflective markers)*. COM, hip and wrist impact velocity (*Force platform)*.
Wang S. et. al. (2020)[58]	Quasi-experimental. n= 195;(72.3±5.3 years).	Chart a recovery rate curve for gait-slips. Investigate and determine the optimal recovery zone.	Slip: was induced by movables platforms, low-friction, in a walkway 7 meters.	-	COM position and velocity *(Force platform)*. Step length, limb support, foot touchdown, recovery foot liftoff and touchdown *(Camera motion capture system and retro-reflective markers).*
Rogers et. al. (2021)[75]	Randomized controlled trials. n=78 MW; four group: (73.6±6.5 years), (73.7±6.3 years), (72.5±7.2 years) and (70.8±4.4 years).	Compare the effects different training prospective falls among older adults.	Waist-pull: Perturbation medial-lateral (ML) was applied by a motorized system.	Single Lateral step. Unloaded limb step (crossover step and medial-lateral step) Collisions between the limbs.	Number of recovery steps, step length, step time, step speed and walking speed. *(An instrumented walkway system).*

Batcir et. al. (2018)[76]	Observational. n=47; OA (≥70 years) and YA (age range 20-30 years).	Valuate the inter-observer reliability and agreement of balance recovery responses, kinematic parameters of stepping, and step threshold.	Surface-translation: the perturbation was applied by a mechatronic device. A computer program provides unexpected surface translation in different directions.	Unloaded leg sidestep. Loaded leg sidestep. Cross-over step. Leg collision. Leg abduction. Multiple steps.	Step initiation, step initiation phase, foot-contact, first recovery stepping duration, first step length, multiple-step duration, multiple-step length and COM distance *(Motion capture system, video camera and reflective markers).*
Werth et. al. (2021)[29]	Quasi-experimental. n=97 MW; YA (24 ±4 years), middle-aged (52 ± 5 years) and OA (72 ± 5 years).	Assess whether volitional step can discriminate between individuals with single or multiple stepping after loss of balance.	Tether-release method: the perturbation was applied with a pneumatic release system.	Single step Multiple steps	Reaction time, swing time, maximal step velocity, step length, rate of increase in BoS foot take-off and foot touchdown. *(Camera motion capture system and retroreflective marker)*
Wang S. et. al. (2019) [63]	Quasi-experimental. n=112 MW. (67.7 ± 5.9 years)	Develop a model based on healthy older adults' normal gait pattern to accurately predict fall-risk following an unexpected.	Slip: was induced by movables platforms, low-friction, in a walkway 7 meters.	Forward Slip.	Step length, step width, gait speed, COM *(Retroreflective marker, camera motion capture system and force platform).* Angle and velocity *(Retroreflective marker, camera motion capture system).*
Wojcik et. al. (1999)[30]	Quasi-experimental. n=20 M; YA (mean age 25 years) and OA (mean age 73.4 years).	Examine the single-step balance recovery capabilities of older women.	Tether-release method: the perturbation was applied with a lean-control cable.	*Forward step:* -Single step -Multiple step failure	Reaction time, weight transfer, step time, step length and step velocity *(Optoelectronic motion analysis system and infrared diodes).*
Batcir et. al. (2020) [37]	Quasi-experimental. n=83 MW; non-faller (79.6±5.1 years), one-time fallers (78±5.5 years) and fallers (78.5 ± 4.7 years).	Investigate the differences in balance recovery to a lateral balance loss between non-fallers, recurrent fallers, and older adults who had fallen only once.	Surface-translation: the perturbation was applied by a system of perturbation multi-directional unannounced underfoot perturbations.	Single step. Multiple-step. Loaded leg sidestep. Unloaded leg sidestep. Crossover step. Leg abduction.	Step initiation duration, step duration, step length, COM path displacement *(Ariel Performance Analysis System, video camera and reflective markers).*

Crenshaw et. al. (2018)[42]	Quasi-experimental. n=112 W; (76.6 ±7.5 years).	Quantify the relationships between established measures of fall risk and compensatory stepping thresholds in older adult women.	Walking a walkway 7.5meters. Trip: obstacle crossing. Disturbances with a computer-controlled treadmill.	Obstacle crossing. Anterior or posterior single-stepping. Anterior or posterior multiple-stepping.	Gait: gait speed, stride time, stride in double-support and step width *(Retro-reflective markers, motion analysis system and video camera).* Trip: Peak lateral speed COM during crossing step and crossing step *(Retro-reflective markers)*.
Carbonneau et. al. (2014)[40]	Quasi-experimental. n=52 MW; YA (32.4±7.7 years), middle-aged (54.3±5.6 years) and OA (75±5.8 years).	Determine the effects of age and loss of balance direction on the threshold of balance recovery, in younger, middle-aged and older adults.	Tether-release method: the perturbation was applied with a lean-control cable.	-	Reaction time, weight transfer time, step time, mean and maximum step velocity, step length, step width and step height *(Optoelectronic position sensors and markers).* Before liftoff and after touchdown *(Force platforms).*
Lockhart. et. al. (2005)[28]	Quasi-experimental. n=42 MW; YA (22.6±2.1 years), middle-age (46.9±13.6 years) and OA (75.5±6.8 years).	Investigate the process of initiation, detection, and recovery of inadvertent slips and falls.	Slip with vinyl tile. The vinyl tile surface was covered with motor oil to reduce the coefficient of friction.	-	Step length, Step length index, heel contact velocity, velocity of the whole-body COM and friction demand. *(Performance Analysis System, video camera and reflective markers).*
Yungher et. al. (2012)[67]	Quasi-experimental. n=20 MW; (Mean age 72.8 years).	Measure the changes over time of protective stepping within a single session of repeated lateral perturbations in older adults.	Waist-pull: the perturbation was applied by a perturbation system in the right and left lateral direction.	Lateral sidestep. Unloaded crossover step by front body. Unloaded crossover step by behind the body. Unloaded step medially.	Number of steps, number strategies steps, number of collisions, medial-lateral distance, onset and cessation step time, mean velocity, leg length. *(Reflective markers and camera motion analysis system)*
Grabiner et. al. (2012)[71]	Randomized controlled trial. n=52 W; training group (65.9 ± 7.8 years) and control group (58.8±4.7 years).	Determine the extent to which a task-specific training protocol decreased the number of falls by middle-age and older women after a laboratory-induced trip.	Surface-translation: the perturbation was induced by a microprocessor-controlled, stepper motor-driven treadmill. Trip: the foam obstacle has a 5cm high.	-	During initial acceleration phase, during the constant velocity phase, during the deceleration phase, trunk flexion angle and velocity, and recovery step length. *(Reflective markers and motion capture system).*

Rogers et. al. (2001)[57]	Quasi-experimental. n=50 MW; adults (31±7 years) and OA: non-faller (71±5 years) and faller (74± 8 years).	Investigate whether the protective stepping for balance recovery is altered with age and history of falling.	Waist-pull: the perturbation was applied by a motor-driven pull system.	-	First-step liftoff time, step distance, step duration, COM displacement and velocity *(Motion analysis system, video camera and reflective markers).*
Ochi et. al. (2014)[52]	Quasi-experimental. n=29 W; non-faller (81.4 ± 3.4 years) and faller (82.8 ± 4.5 years).	Compare leg muscle activation patterns during recovery steps after a forward fall between.	Tether-release method: the perturbation was applied with the release of the tether.	-	Step length and knee angle *(markers in the floor and a video camera).* Lift-off phase, step phase and stance phase, onset time and time to first peak *(load cell, foot switch and video data).*
Pijnappels et. al. (2005) [55]	Quasi-experimental. n=23 MW; YA (27.1± 4.3 years) and OA (67.6±2.7 years).	Investigate whether older adults react less than young adults during the primary phase of recovery after tripping.	Trip: walking over a platform. A force plate was mounted in the platform and 21 aluminum obstacles were hidden over a total distance of 1.5 m.	Elevating strategy. Lowering strategy.	*Kinematic*: heel strike, toe-off, obstacle foot contact. *Time*: stance phase, swing phase and double support duration. *Clearance:* stride length, hip height and hip displacement. *(Video camera and infrared-light markers).*
Okubo et. al. (2019)[73]	Randomized controlled trial. n=44 MW; an intervention group (73.3±5.9 years) and control group (70.8±4.9 years).	A reactive balance training program with slips and trips would reduce perturbation-induced falls in older adults.	Slip and trip: the perturbation was applied in a 10m walkway. Both perturbations were concealed. Slip was induced by a movable tile on two hidden rails. Trip were induced using by a foot sensor.	*Trip:* Elevating strategy. Lowering strategy. Slip: Backward stepping. Forward stepping.	*Gait:* speed, cadence and step length. *Trip and slip:* MoS, COM position, step length, step height and trunk degree. In addition, foot contact slip, slip speed and slip distance *(Camera motion capture system and reflective markers).*
Pavol et. al. (2001)[68]	Quasi-experimental. n=79 MW; (72±5 years).	Identify the mechanisms whereby healthy older adults fell following an induced trip.	Trip: the perturbation was induced using a concealed, pneumatically driven, metal, mechanical obstacle.	Elevating strategy or reaching strategy. Lowering strategy.	Trunk angle, hip velocity, time trip, step length, stride length, foot toe-off, time ground contact, step duration and ankle velocity *(Camera motion capture system and reflective markers).*
Bosquée et. al. (2021)[39]	Quasi-experimental. n=44; YA (24 ± 3 years), middle-aged (53 ± 5 years) and OA (72 ± 5 years).	Examine the relationship between the stability recovery performance during lean-and-release task and a tripping-task	Tether-release method: the perturbation was applied by a custom-built pneumatic break and release system. Trip: the perturbation induced using a	*Tether-release method* Single step. Multiple steps.	MoS, foot touchdown and BoS *(Camera motion capture system and reflective markers).*

		on a treadmill among adults.	custom-built pneumatic perturbation system.		
Mille et. al (2013)[22]	Quasi-experimental. n=75 MW; YA (23.5±3.2 years) and OA: non-faller (72.5±5.9 years) and faller (75.2±7.8 years).	Determine the protective stepping response evoked by different directions of externally applied waist-pull perturbations of balance and to identify the changes in stepping attributable to aging and in relation to the risk of falling.	Waist-pull: the perturbation was applied by a motor-controlled waist-pull system.	Single step or forward step.<br>Multiple steps.<br>Lateral Loaded step.<br>Lateral Unloaded step.<br>Collision step.<br>Unloaded mediolateral steps.<br>Crossover step.<br>Collision step.	Number of steps, onset time, step duration, step clearance, global step length *(Camera motion analysis system, reflective markers).*
Young et. al. (2013)[66]	Quasi-experimental. n=39 MW: fallers (73.4±1.1 years) and non-fallers (74.6±1.6 years).	Determine how leg preference affected balance recovery strategies following a perturbation balance.	Waist-pull: the perturbation was induced by a motor waist-pull system.	Lateral sidesteps.<br>Unloaded crossover step. or crossover step.<br>Medial sidestep.	Numbers and types of steps. (*Observation, camera motion capture system and reflective markers).*
Rieger et. al (2020)[74]	Randomized controlled trial. n=30 MW; intervention group (70.33±3.99 years) and control group (71.67±4.96 years).	Investigate the effects of a single session gait training with perturbations in AP direction on ML gait stability and assessed whether these effects are retained.	Surface-translation: the perturbation was applied in a 3-D instrumented dual-belt treadmill.	Crossover step.	Step time, stance time, swing time, step length, step width trunk velocity and COM *(Camera motion capture system and reflective markers).*
Carty et. al (2012)[41]	Quasi-experimental. n=151 MW. (71.6±4.6 years)	Investigate balance recovery in the anterior-posterior direction exhibited by older adults.	Tether-release method: the perturbation was applied through the disengagement of an electromagnet.	Single step.<br>Multiple steps.	MoS, BoS and velocity COM (*Force platform).* Toe off duration and foot contact duration *(Camera motion capture system, reflective markers).*
Graham et. al. (2015)[45]	Quasi-experimental. n=117 MW. (72±4.9 years)	Identify the biomechanical factors that distinguish between older adults who con recover with a single step forward lean.	Tether-release method: the perturbation was applied through the disengagement of an electromagnet located in-series with the cable.	Single step.<br>Multiple steps.	Step length, step time, trunk angle and angular velocity at toe off *(Camera motion capture system, reflective marker).* Foot contact *(Platform).*

Mansfield et. al (2010)[72]	Randomized controlled trial. n=30 MW; intervention group (70.3±4.7 years) and control group (69.1±3.8 years).	Evaluate a perturbation-based balance training program.	Waist-pull and surface-translation: the perturbations were applied with an unpredictable system of support surface motion (motion platform) and cable pulls.	Multiple steps. Lateral step. Foot collisions.	Frequency of multiple-step, lateral step and collisions during lateral step reactions, and step distance. *Reaction time (Motion capture system, video camera and reflective markers).* Foot-off and foot-contact *(Force platforms).*
Pavol et. al. (1999)[54]	Quasi-experimental. n= 79 MW. (72±5 years).	Determine the proportion of falls that resulted from trip induced during gait.	Trip: the perturbation was induced using a concealed pneumatically, driven metal, mechanical obstacle.	-	Step time, step length, stride length, step width and average trunk flexion angle. *(Motion capture system and reflective markers).*
Singer et. al (2015)[60]	Quasi-experimental. n= 20; YA (25±5 years) and OA (67±6 years).	Determine the age-related biomechanical differences during recovery from posterior and anterior perturbations.	Tether-release method: the perturbation was applied through the disengagement of an electromagnet.	Single step Multiple step	Reaction time, step time, recovery time *(Camera motion capture system and reflective markers).* Step length and peak COM velocity *(Reflective markers and plate platform*).
Yang et. al. (2014)[65]	Quasi-experimental. n= 187 MW; (71.9±5.1 years).	Evaluate the degree to which these stability measurements could predict an impending fall.	Slip: the perturbation was applied by a sliding device along a 7m walkway. The device consisted of two low frictions.	-	Step length, step width and step time. *(Camera motion capture system and retro-reflective markers)*
Batcir et. al. (2022)[24]	Quasi-experimental. n= 84 MW; (79.3±5.2 years).	Explore whether different kinematic patterns and strategies occur in the first recovery single-step and multiple-step.	Surface-translation: the perturbation was applied by motor driven treadmill device. The treadmill provided a right or left unannounced surface translation.	Single-step. Multiple-step. Loaded-leg sideway stepping. Unloaded-leg sideway stepping. Cross-over stepping. Hip Abduction. Leg Collisions.	Step initiation, step duration, step length, step velocity, total recovery duration, recovery step path length and total estimated COM. *(Motion capture system, retro-reflective markers and video camera)*
Luchies et. al. (1999)[48]	Quasi-experimental. n= 24; YA (21±2.5 years) and OA (68±4.1 years).	Investigate if performance on an involuntary step task was comparable to	Waist-pull: the perturbation was produced using electronically released weight and cable system.	-	Liftoff time, landing time, time, weight transfer time, step duration, step length and step height *(Landmark motion, optoelectronic motion analysis system).*

		performance on a voluntary step task.			
Owings et. al. (2001)[53]	Quasi-experimental. n= 79 MW. (72±5 years).	Determine if the mechanisms biomechanical that contributed to failed recoveries in the treadmill would be the same by from a trip.	Surface-translation: the perturbation was applied by a motorized treadmill.	-	Reaction time, step length, recovery step, onset, toe-off and ground contact, step length, trunk angle at onset, toe-off and ground contact. And trunk velocity at toe-off and ground contact *(Camera motion capture system and reflective markers).*
Thelen et. al. (1997)[61]	Quasi-experimental. n= 20; YA (24.3 mean years) and 10 OA (mean age 71.3 years).	Measure the largest forward body lean angle that could regain standing balance by taking a single, rapid step.	Tether-release method: the perturbation was induced by releasing the lean-control cable.	-	Reaction time, transfer time, step time, step length and step velocity *(Optoelectronic motion capture system and diodes markers).*
Cronin et. al. (2013)[43]	Quasi-experimental. n= 81 MW. (70±3 years).	Compare the timing and magnitude of activation of the major leg muscles during balance recovery.	Tether-release method: the perturbation was induced by the disengagement of an electromagnet.	Single step. Multiple steps.	Step length, step time, ankle and knee angle *(Motion capture system, retro-reflective markers and video camera).*
Troy and Grabiner (2005)[62]	Quasi-experimental. n= 13 MW. (72±5 years).	Examine how older adults restore dynamic equilibrium after three types of large postural disturbances.	Tether-release method: the perturbation was induced by the disconnection of an electromagnet. Surface-translation the perturbation was induced by a motorized treadmill. Trip: the perturbation was induced by a pneumatically powered obstacle.	-	Maximum step height, step length of the leading and trailing limbs, peak vertical velocity, and peak horizontal velocity of the leading and trailing feet *(Reflective markers).*
Mille et. al. (2005) [50]	Quasi-experimental. n=20 W; YA (24±1.4 years) and OA (73.3±6.3 years).	Determine age-related differences in protective stepping behavior in response to lateral waist-pull perturbations of postural balance.	Waist-pull: the perturbation was applied by a motor-driven waist-pull system.	Single step. Multiple steps. Loaded sidestep. Unloaded medial step. Unloaded crossover step. Collisions between feet.	Number of steps, step strategies and collisions between feet. Onset and end step, step displacements, step clearance and trunk angular displacements *(Camera Motion capture system and reflective markers).*

Dijkstra et. al. (2015)[44]	Quasi-experimental. n=25 MW; OA (68±7 years) and YA (28±4 years).	Determine whether young and older adults exhibit similar improvements in compensatory stepping over repeated exposure to external perturbations.	Surface-translation the perturbation was induced by surface translations.	Side-step. Cross-over step.	First step latency, first step length, number of steps and identification of lateral step strategy *(Motion capture system and reflective markers).*
Nagano et. al. (2015)[51]	Quasi-experimental. n=15;(72.5±4.8 years).	Understand how older adults control lower limb stepping responses to preserve balance following unanticipated forward falling.	Tether-release method: the perturbation was applied by disconnection to the electromagnet system.	Single-step. Multiple-steps.	Initial reaction, foot contact, step length, step width, step velocity, COM velocity and MoS *(Motion capture system, video camera, reflective markers).*
Kim et. al. (2013)[47]	Quasi-experimental. n= 41 MW; YA (22.7±3.35 years) and OA (68±7.19 years).	Assess the effects of perturbation type (MP vs LP) and advancing age on recovery step and trunk Kinematics.	Perturbing shoes: the perturbation was applied by instrumented sandals. Each sandal has two electronically-controlled hinged flaps concealed.	-	Walking speed and step width *(Optoelectronic camera system and infrared-emitting diode markers).*
Borrelli et. al. (2019)[38]	Quasi-experimental. n= 48 MW; (72.7±5.6 years).	The balance stability of the different evoked first step reactions and the stability of single and multistep strategies of older adults to lateral waist-pull perturbations.	Waist-pull: the perturbation was applied by the waist-pull system. The system includes a stepper- motor driven linear drive table, cable and belt connection, in-line load cell, position transducer, floating electromechanical latch, and spring pre-tensioner.	Laterals sidestep. Unloaded sidesteps: Cross-over step to the front. Cross-over step to the back. Medial sidestep. Multiple steps. Inter-limb collisions. Single step.	MoS, step initiation and step termination step length, swing time, first step BoS-COM and COM velocity *(Motion capture system and reflective markers).*
Borrelli et. al. (2021)[23]	Quasi-experimental. n= 71 MW; (72.7±5.5 years).	Compare the space-temporal stepping parameters and MoS between a single lateral sidestep and each step of a two-step (unloaded sidesteps - followed by a	Waist-pull: the perturbation was applied by the waist-pull system. The system includes a stepper- motor driven linear drive table, cable and belt connection, in-line load cell, position transducer, floating	Laterals sidestep. Unloaded sidesteps: Cross-over step to the front. Cross-over step to the back. Medial sidestep.	Step unloading onset, step loading, time between limb unloading onset and loading *(Motion capture system, reflective markers).* COM displacement at step loading, and COM velocity at step loading *(Motion capture system, reflective*

		lateral sidestep) protective stepping sequence.	electromechanical latch, and spring pre-tensioner.	Multiple steps. Inter-limb collisions. Single step. Single lateral sidestep. Unloaded sidesteps followed by a lateral sidestep.	*markers).* Lift-off and touch down *(Force platform).*
Shulman et. al. (2019)[59]	Quasi-experimental. n= 34 MW; OA (75.6±5.3 years) and YA (21.7±2.6 years).	Discern age related alterations in proactive and reactive Dynamic postural control of gait initiation.	Perturbations of the support surface: the perturbation was applied by force plates. The force platform was mounted on top of a custom robotic platform.	-	Toe-off, heel contact, step length, step width, step time and whole-body COM velocity *(Camera motion capture system and reflective markers).*
Allin et. al. (2020)[70]	Randomized controlled tried. n= 34 MW; (age range 61-75 years).	Evaluate the effects of perturbation-based balance training targeting slipping and tripping on laboratory induced slips and trips.	Slip: the perturbation was induced by spreading a thin layer of vegetable oil over a section of the walkway. Trip: the perturbations was induced using a tripping obstacle that was initially concealed and level with the walkway.	*Trip* Elevating strategy. Lowering strategy.	Gait speed, step length and minimum toe clearance. *Slip and trip:* onset, touchdown, minimum hip height and MoS at touchdown. Slip: slip end, peak slip speed, distance and COM velocity relative. Trip: trunk angle at touchdown, recovery step length and stepping strategy *(Camera motion capture system and reflective markers).*
Graham et. al. (2014)[46]	Quasi-experimental. n= 15 M; OA (70±3.9 years) and YA (34±2.6 years).	Determine the muscular contributions to the stepping phase of recovery from forward loss of balance with a single step, and multiple steps.	Tether-release method: the perturbation was applied through the disengagement of an electromagnet located in-series with the cable.	Single steps. Multiple steps.	Step length, step time, toe-off, mind swing foot contact and reaction force *(Motion capture system, reflective markers)*
Mansfield et. al. (2009)[49]	Quasi-experimental. n= 40 MW; YA (age range 22-28 years) and OA (age range 64-79 years).	Determine if previously reported age-related differences in change-in-support reactions are dependent on perturbation method, under conditions where other confounding factors are controlled.	Surface-translation: the perturbations were delivered via rapid horizontal translation of the computer-controlled motor-driven motion platform. Cable-pull/ Waist-pull: the perturbations were applied by dropping a weight of subject, using a computer and	-	*Step reaction*: frequency of multiple-step reactions, stepping pattern, frequency lateral step and collisions between the swing and stance limbs *(Motion capture system and reflective markers). Initial step:* onset, foot-off, foot contact, step length, and swing duration *(Motion capture system and reflective markers).*

			electromagnet to control the timing of the drop.		
Lee et. al. (2014)[26]	Quasi-experimental. n= 26; YA (two groups (26.5±3.4 years) - (25.3±4.6 years)) and OA (two groups (69.2±10.2 years) - (71±6.7 years)).	Examined forwards stepping despite considerable bio-mechanical and visual differences between the forwards and backwards directions	Surface-translation: the perturbation was applied by a motorized computer-controlled platform.	-	Step length, foot lift-off and landing step *(Platform conductive strips)*. Step reaction time and angular velocity step *(Gyroscopes and force platform).*
Aprigliano et. al. (2017)[36]	Quasi-experimental. n= 20; YA (24.4±2.5 years) and OA (66.3±5.1 years).	Verify whether aging modifies the intralimb coordination underlying corrective responses induced by unexpected perturbations.	: the perturbation was applied by a split-belt treadmill whose belts can be independently controlled the perturbations.	-	Duration of the stride and stance phase, step length, and width step. *(Motion capture system and reflective markers)*
Nnodim et. al. (2016)[31]	Quasi-experimental. n=8 MW; (72±6.4 years).	Identify if there are interference between the motor and the cognitive after a gait-destabilizing perturbation.	Perturbing shoes: the perturbation was applied by an instrumented shoe. The footwear has two retractable connected a program.	-	Gait sped, step width, step length and step time *(Motion capture system and diode markers)*
Chen et. al. (1994)[21]	Quasi-experimental. n= 48 MW; YA (mean age 23.4 years) and OA (mean age 72.8 years).	Test that neither age, gender, nor available response times affect the strategies used to step over an obstacle appearing with short available response times.	Trip: the perturbation was applied by a virtual obstacle with a light projected onto the 8m walkway.	Short step strategy. Long step strategy.	Pre crossing obstacle and crossing obstacle: step length, step time. Post crossing obstacle: toe distance and heel distance. Step strategy: Number of steps deliberate *(Walkway with conducing surface and computer)*
Bianca Te et. al. (2023) [77]	Observational.<br><br>n= 515 OA (mean age 82.7 years)	Determine the relative importance of falls an absence of stepping responses, versus steps that were inappropriate in size or direction.	Analyzed video footage of falls experienced by residents.	Multiple steps.	Number of steps, direction of the steps, length of the first and second sept. *(Digital video cameras)*
Wang S. et. al. (2023) [69]	Quasi-experimental. n=298 OA (69.6±6.33 years)	Develop prediction models to predict trip-related fallers with different recovery	Trip: the perturbations was induced by an obstacle device which consisted of a hinged metal plate and a pair of	Lowering strategy. Elevating strategy.	*Gait:* gait speed, trunk angle, COM state, step length, gait duration, toe clearance, swing time, and step time. *Joint kinematics*: hip angle, knee angle, and foot angle for both limbs

		strategies using machine-learning methods	electromagnets. Walking in a walkway 7 meters.		*(Motion capture system and reflective markers)*
Tashiro et. al. (2021) [70]	Quasi-experimental. n=W; OA (77.8±6.9 years).	Determine the relationship between protective lateral stepping ability and FOF among older community-dwelling individuals.	Tether-release method: the perturbation was applied through a cable was released suddenly after a random delay ranging.	Single-step.<br>Multiple stepping.<br>Lateral steppers.<br>Crossover steppers.<br>Medial steppers.	Types of steps.<br>*(observation)*

Design Research and Population: (Mean ± Standard Deviation); Men (M); Women (W); Men and Women (MW) Youngers Adults (YA) and Older Adults (OA). Variables and Measuring Instrument: Margin of Stability (MoS); Center of Mass (COM) and Base of Support (BoS).
